# Roles of Health Literacy in Relation to Social Determinants of Health and Recommendations for Informatics-Based Interventions: Systematic Review

**DOI:** 10.2196/50898

**Published:** 2024-03-20

**Authors:** Shwetha Bindhu, Anunita Nattam, Catherine Xu, Tripura Vithala, Tiffany Grant, Jacinda K Dariotis, Hexuan Liu, Danny T Y Wu

**Affiliations:** 1 College of Medicine University of Cincinnati Cincinnati, OH United States; 2 School of Medicine Case Western Reserve University Cleveland, OH United States; 3 University of Cincinnati Libraries Research and Data Services University of Cincinnati Cincinnati, OH United States; 4 Department of Human Development and Family Studies The University of Illinois at Urbana-Champaign Urbana, IL United States; 5 The Family Resiliency Center College of Agricultural, Consumer and Environmental Sciences The University of Illinois at Urbana-Champaign Urbana, IL United States; 6 Department of Biomedical and Translational Sciences The University of Illinois at Urbana-Champaign Urbana, IL United States; 7 School of Criminal Justice University of Cincinnati Cincinnati, OH United States

**Keywords:** health literacy, social determinants of health, SDoH, social determinants, systematic review, patient education, health education, health information, information needs, information comprehension, patient counseling, barriers to care, language proficiency

## Abstract

**Background:**

Health literacy (HL) is the ability to make informed decisions using health information. As health data and information availability increase due to online clinic notes and patient portals, it is important to understand how HL relates to social determinants of health (SDoH) and the place of informatics in mitigating disparities.

**Objective:**

This systematic literature review aims to examine the role of HL in interactions with SDoH and to identify feasible HL-based interventions that address low patient understanding of health information to improve clinic note-sharing efficacy.

**Methods:**

The review examined 2 databases, Scopus and PubMed, for English-language articles relating to HL and SDoH. We conducted a quantitative analysis of study characteristics and qualitative synthesis to determine the roles of HL and interventions.

**Results:**

The results (n=43) were analyzed quantitatively and qualitatively for study characteristics, the role of HL, and interventions. Most articles (n=23) noted that HL was a result of SDoH, but other articles noted that it could also be a mediator for SdoH (n=6) or a modifiable SdoH (n=14) itself.

**Conclusions:**

The multivariable nature of HL indicates that it could form the basis for many interventions to combat low patient understandability, including 4 interventions using informatics-based solutions. HL is a crucial, multidimensional skill in supporting patient understanding of health materials. Designing interventions aimed at improving HL or addressing poor HL in patients can help increase comprehension of health information, including the information contained in clinic notes shared with patients.

## Introduction

### Overview

In recent decades, medical providers, health systems, and legislators have prioritized increasing patient access to health information. For example, the 21st Century Cures Act mandates that patients must have access to their electronic health records, including clinic notes, in a rapid and convenient manner [1]. However, clinic notes and other health information can contain jargon that is difficult for patients to comprehend, reducing the utility of health information sharing. The Healthy People 2030 initiative, sponsored by the US Department of Health and Human Services, aims to address this issue by increasing patient comprehension of health information received from providers and web-based sources, such as their electronic health records [2].

A key part of health information comprehension is health literacy (HL), the ability to understand, contextualize, and make well-informed decisions based on health information [3]. Reducing HL gaps is crucial to meeting the goals set forth by Healthy People 2030 and maximizing the benefits of the 21st Century Cures Act.

### Health Literacy

Having high HL correlates with greater shared decision-making between patients and physicians and promotes positive health outcomes because patients can better comprehend and act on the health information they receive [4]. Healthy People 2030 distinguishes between two dimensions of HL: personal, as previously described, and organizational [2]. Organizational HL holds health care systems and providers accountable for providing their patients with comprehensible health information to make informed decisions. This newer understanding of HL raises questions about how HL fits into the public health framework addressing disparities in health comprehension.

### Social Determinants of Health and Health Literacy

Social determinants of health (SDoH) are nonmedical social and economic factors that fall into the following 5 domains: economic stability, education, health care and access quality, neighborhood and built environment, as well as social and community context [5,6]. SDoH affects health status and outcomes, and it can generate health disparities between population groups by influencing patient behavior and organizational responses. These determinants are also distinct from social factors or needs that exist at the individual level and instead exist as community- or population-level barriers [7-9].

**Figure 1:**
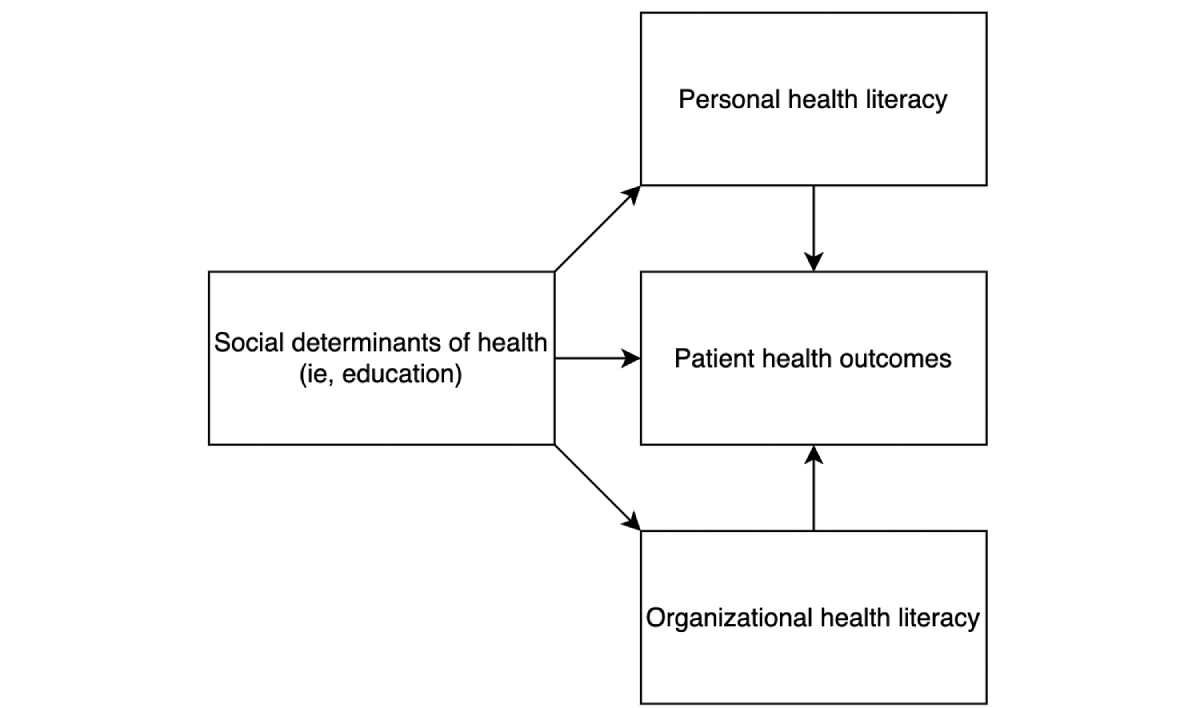
Relationships characterized by the influence of social determinants of health on health literacy (personal and organizational), subsequently impacting health outcomes.

HL has been categorized in different sources as an SDoH itself and as a midstream consequence of SDoH that can impede or improve patient interactions with health care institutions and health outcomes (ie, vaccination status and screening utilization) [10-12]. For example, a study by Schillinger et al [13] proposes that a higher education level improves HL, which was associated with better glycemic control among patients with diabetes. This is a unidirectional characterization of the relationship between SDoH, HL, and health outcomes, depicted in Figure 1.

However, this may be an oversimplification. HL can evolve through continued exposure to health environments and interventions at the personal and organizational levels [14]. Moreover, even patients with high HL can struggle with comprehension in different contexts. Therefore, this relationship warrants further investigation, as there is a lack of systematic literature analyses that macroscopically evaluate how SDoH and HL are related across different SDoH domains [15]. Understanding the nature and role of HL in interactions with SDoH can also indicate the most effective approach to designing HL-targeting interventions for patients who struggle to understand health information.

### Objectives

Due to the literature gap in examining the complex relationship between HL and SDoH, we aimed to conduct a systematic literature review to (1) understand this relationship and (2) recommend informatics-based interventions to address low HL among patients.

## Methods

### Search Strategy

We systematically reviewed literature in PubMed and Scopus, two major biomedical and social science literature repositories. The initial database searches were conducted on June 22, 2020. The review followed the PRISMA (Preferred Reporting Items for Systematic Reviews and Meta-Analyses) 2009 guidelines to understand the relationship between HL and SDoH [16,17].

The search terms used were “health literacy” AND “social determinants of health.” After filtering for non-English articles and articles without abstracts, the remaining 281 articles were compiled in a Microsoft Excel sheet with their title, author, publication year, DOI or PMID, and abstract.

### Screening Process

Two researchers (SB and CX) independently screened 281 papers by title and abstract and used the following exclusion criteria: (1) HL is a minor factor in the article; (2) the article is not an empirical study; (3) the article focuses on HL measurement tool development or evaluation; (4) the paper does not examine HL in relation to SDoH; (5) no abstract is available; and (6) the paper is not written in English.

Each researcher independently gave the article a score of “1” for inclusion or “0” for exclusion. The scores were summed; articles scoring “2” were automatically included, and those scoring “0” were excluded from the full article eligibility review. Disagreements (any papers with a total score of “1”) were resolved by the authors after the initial screening. The process was repeated for the full-article eligibility review and subsequent reference screening from the included full articles. Reference screening was a precautionary step to ensure the inclusion of articles that may not have been included in the initial database search. Original exclusion criteria were consistently used.

### Quality Assessment

Before the information extraction, all included articles were assessed by 2 researchers (SB and TG) for study quality. Using the Agency for Healthcare Research and Quality (AHRQ) guidelines, separate quality assessments were developed for each type of study included in the review—observational studies and randomized clinical trials (RCTs) [18]. Domains included in both study types were study questions, population, interventions, outcome measurement, statistical methods, results, discussion, and disclosure of funding or sponsorship. Domains evaluated in the RCT assessment also included blinding and randomization.

The reviewers created a 3-point scoring system for the quality assessment. Articles were rated by 2 team members (SB and TG) with scores of “good,” “fair,” and “poor” for each domain and assigned numerical values of 2, 1, and 0, respectively, as per the AHRQ guidelines [18]. Values were averaged and translated back to a rating of “good” (1.50 or higher), “fair” (1-1.49), and “poor” (0-0.99).

### Information Extraction

Based on quality assessment results, 43 papers were included for information extraction. Four researchers (CB, CX, AN, and TV) extracted data for the following PRISMA-based criteria: title, author, article ID, year published, location, study design, sample demographics, results, and limitations [16]. To answer the research questions, information specific to SDoH focus, HL measurement, and health outcomes was collected. The information extraction sheet is attached as Multimedia Appendix 1.

### Quantitative and Qualitative Data Analysis

Extracted data were analyzed both quantitatively and qualitatively. Location, year of publication, and study design were statistically summarized. AHRQ guidelines were used to categorize studies as RCT, cross-sectional, and qualitative designs, with the last two being types of observational studies [19].

Qualitative analysis was conducted in 2 steps. First, a narrative synthesis of the chosen articles summarized the relationships between SDoH and HL. Narrative synthesis involves analyzing the data from systematic reviews to create textual explanations of observed patterns or trends rather than relying solely on statistical data. This involves developing textual descriptions of the data by extracting key information pertinent to the research question (ie, methods used or results) and exploring commonalities and differences between and within studies (ie, through visually mapping relationships) [20]. These methods were also used in a systematic review previously published by the authors [21]. The included articles were classified by SDoH domains they addressed, per the 5 domains defined by Healthy People 2030: economic stability, education, health care access and quality, neighborhood and built environment, as well as social and community context. Then, information extraction data from article results and discussion sections were used to define roles for HL. Finally, a theme visualization was conducted that plotted HL roles against publication year to understand how HL perception has evolved.

In the second step, lessons learned were summarized regarding HL roles, again using the results and discussion sections. From these same sections, the authors then extrapolated possible interventions that use HL to improve patient comprehension of health information.

## Results

### Literature Search Results

**Figure 2:**
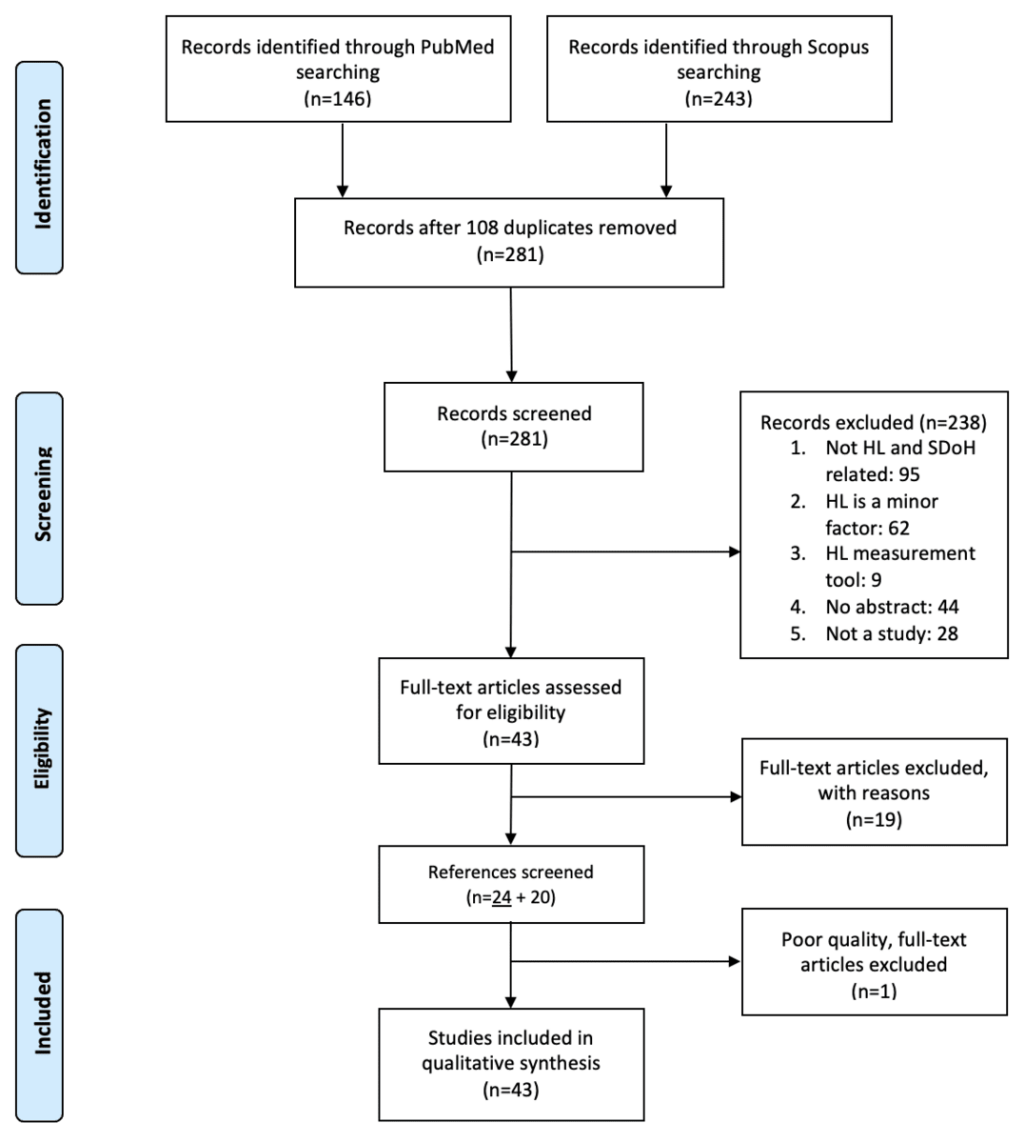
The PRISMA (Preferred Reporting Items for Systematic Reviews and Meta-Analyses) diagram of articles from the PubMed and Scopus search. A total of 24 articles (underlined) were included from the first database search.

The PubMed and Scopus searches yielded 389 articles, resulting in 281 unique articles (Figure 2). After screening titles and abstracts, 43 articles remained for full-text eligibility assessment. Not discussing HL and SDoH together (n=95) was the largest cause for exclusion. Other papers were excluded because HL was not a substantial focus of the paper (n=62). A total of 19 articles were excluded from the full-text eligibility, once again for a minor focus on HL. References of the remaining 24 articles were screened for inclusion, yielding 20 additional articles. After the quality assessment, 1 low-quality article was excluded. Information extraction and narrative synthesis were conducted on a final sample of 43 articles.

### Quantitative Analysis

The final 43 articles were analyzed for study location, year of publication, role of HL, and study design (Table 1). A total of 14 (32.6%) studies took place in North America (12 in the United States and 18 in Europe). All articles from South America originated in Brazil (n=4). Publication year trends revealed an increased focus and discussion of the topic in recent years, with 90.7% (n=39) of the articles being published after 2010. Most articles (n=40, 93%) had a cross-sectional design and used surveys, while 4.7% (n=2) used a qualitative design with semistructured interviews and focus questions to assess SDoH and HL.

**Table 1:** Summary of articles included in the literature review (N=43).

Category		Values, n (%)
**Study location**		
	Europe	18 (41.9)
	North America	14 (32.6)
	Asia	6 (14)
	South America	4 (9.3)
	Australia	1 (2.3)
**Year published**		
	2006-2009^a^	4 (9.3)
	2010-2013	9 (20.9)
	2014-2017	15 (34.9)
	2018-2021	15 (34.9)
**Study design**		
	Cross-sectional	40 (93)
	Qualitative	2 (4.7)
	Randomized controlled trial	1 (2.3)

^a^The search was not limited to 2006 for publication year; this was the earliest date among the 43 articles.

### Qualitative Analysis

#### Narrative Synthesis

The narrative synthesis generated 4 roles for HL in relation to SDoH (Table 2). Most of the articles discussed multiple SDoH domains, but all 43 articles discussed education access and quality [5].

The most common categorization of the HL role was as a “result of SDoH” (n=23), followed by “modifiable SDoH” (n=14), and finally, as a “mediator of SDoH” (n=6). HL can be a “result of SDoH” (n=23), which suggests that SDoH domains contribute to HL levels and that it is a downstream variable [22-44]. As mentioned, 14 studies identified HL as a “modifiable SDoH,” where they identified HL as an SDoH, often citing the World Health Organization’s categorization of it; these studies suggested that HL can be improved through interventions and is actionable at multiple levels [14,45-57]. Finally, the articles that categorized HL as a “mediator of SDoH” (n=6) discussed how HL is an intermediary between other SDoH domains, such as educational attainment or economic stability, and that high HL levels can compensate for lower domain levels that compromise positive health outcomes [58-63]. Occasionally, the same paper would suggest multiple roles for HL (eg, an article’s Results and Discussion sections would inform both modifiable and mediatory roles for HL), but the most prominent relationship that appeared was used to categorize each article.

**Figure 3:**
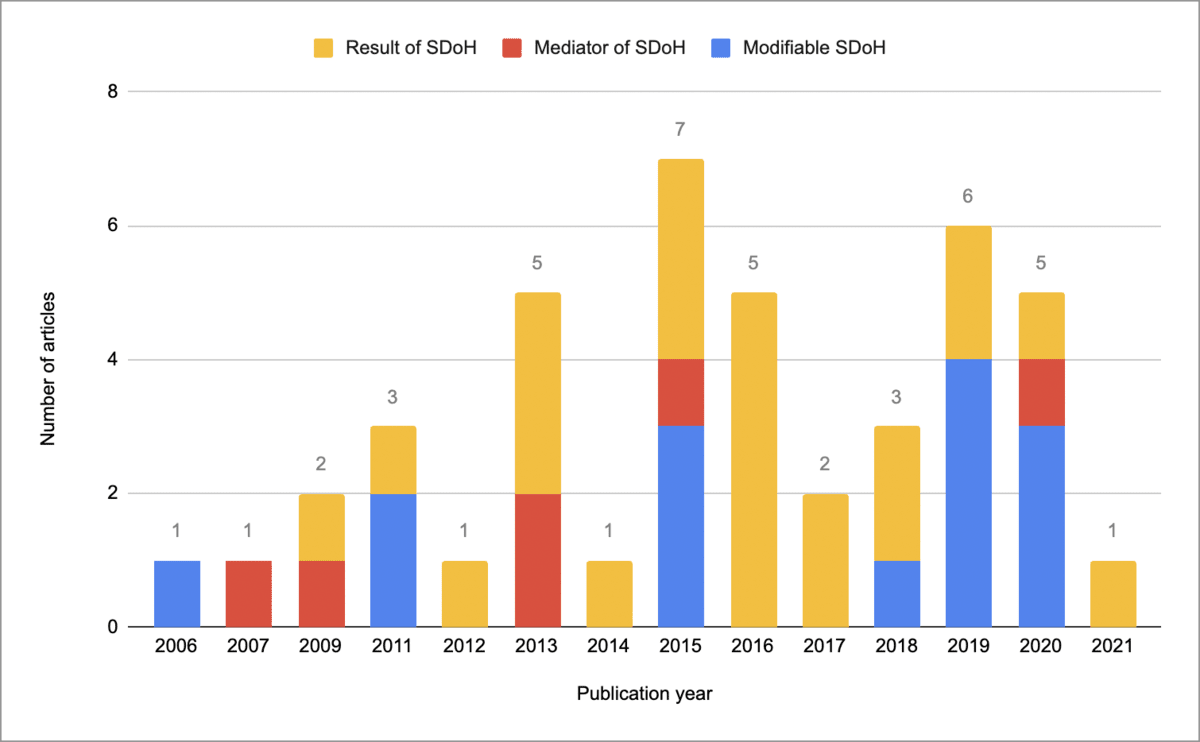
Theme visualization of the evolution of health literacy roles over time. SDoH: social determinants of health.

These 3 roles were plotted against the years of publication in Figure 3. In the few HL-focused articles published before 2010, HL was recognized as having a variety of roles, but only 1 article identified it as a modifiable SDoH. In the next 5-year period, being a result of SDoH was the most common role assigned to HL. In 2013, a total of 5 out of 7 articles identified HL as being a result of SDoH. As the number of published HL-focused articles increased in subsequent years, being a result of SDoH remained the most consistent and most prominent role assigned to HL to appear across all articles. Nevertheless, there has been increasing recognition of HL as a modifiable SDoH in the years 2015, 2018, and 2020, further cementing HL’s multidimensional nature.

**Table 2:** Summary of narrative synthesis themes.

Category		Values, n (%)
**SDoH^a^ domain^b^**		
	Education access and quality	43 (100)
	Economic stability	38 (88)
	Health care and access quality	11 (26)
	Social and community context	11 (26)
	Neighborhood and built environment	9 (21)
**HL^c^ role**		
	Result of SDoH	23 (53)
	Modifiable SDoH	14 (33)
	Mediator of SDoH	6 (14)

^a^SDoH: social determinant of health.

^b^Most of the articles included more than 1 SDoH domain they studied.

^c^HL: health literacy.

#### Lessons Learned

**Figure 4:**
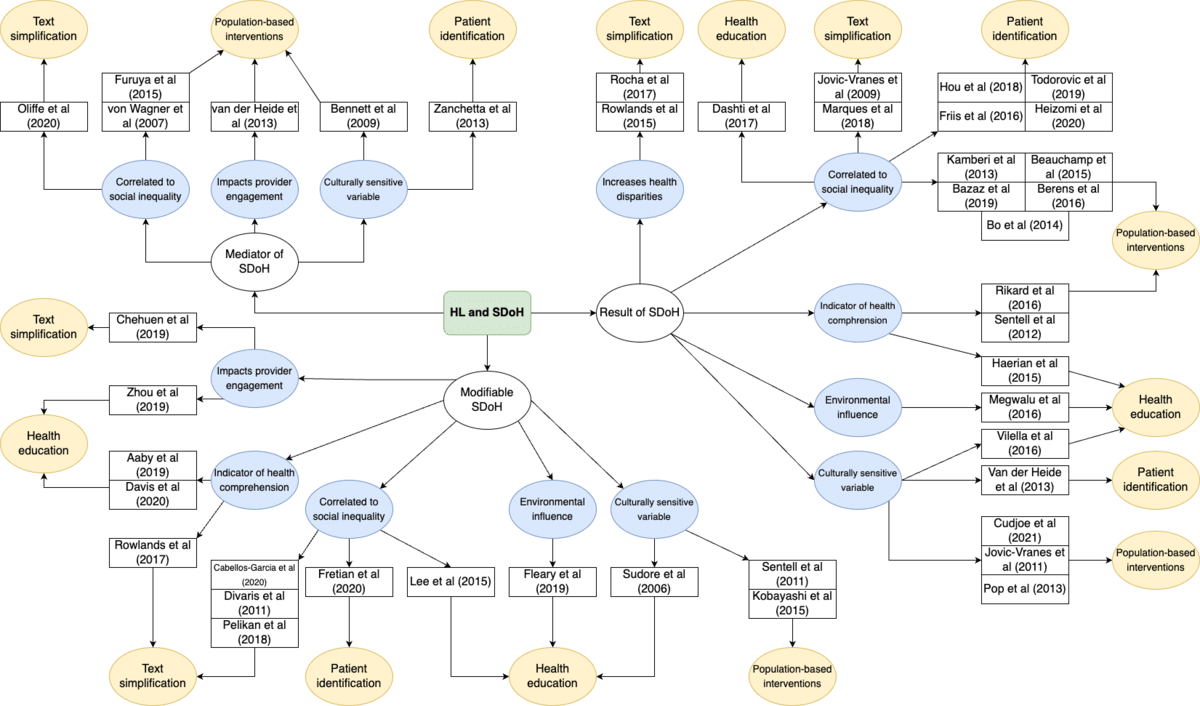
Idea map of health literacy (HL) roles, lessons learned, and article breakdown. Each role (white oval) is broken down into lessons learned (blue oval) and then into the article title and author (white rectangle) and possible intervention focus (yellow oval). SDoH: social determinants of health [14,22-63].

In addition to analyzing articles for the HL role, each article was further examined to determine details about the nature of the relationship between HL and SDoH. These were titled “lessons learned.” Figure 4 [14,22-63] shows an idea map that organizes articles by the role of HL and lessons learned. Although most of the articles are cross-sectional and do not always draw a causal relationship between Hl and SDoH, the authors of the articles nevertheless offer hypotheses on factors influencing HL or how it interacts with SDoH and health outcomes.

#### HL as a Result of SDoH (n=23)

Being a result of SDoH was the most frequent role identified for HL. These articles characterized HL as being associated with, influenced by, or resulting from other SDoH. All articles addressed that a higher level of education, such as high school graduation, had implications on HL levels [22-44]. Bazaz et al [22], Berens et al [24], and Rocha et al [39] have suggested that HL is developed through interactions with health care due to age and disease condition, and more interaction with health care over time leads to an improvement in HL. Hou et al [31], Jovic-Vranes et al [32], Kamberi et al [34], and Todorovic et al [41] also note that lived environments have an important role in HL development. Kamberi et al [34] argue that rural versus urban environments influence SDoH, such as health care access and quality, thereby, impacting HL development [34]. Beauchamp et al [23], Berens et al [24], Cudjoe et al [26], and Sentell et al [40] observed that HL is also influenced by the patient’s primary language, especially if the patient’s primary language is different from the language of the health system. Bo et al [25] and Pop et al [37] elaborated on the relationship between education and HL; they found that lower levels of language proficiency and self-perceived health can indicate lower HL. Heizomi et al [30] and Dashti et al [27] notice gender disparities in HL among students in Iran, with the latter observing that cultural differences encouraged technology access for men at a younger age, leading to higher HL levels among men compared to women [27,30].

#### HL as a Modifiable SDoH (n=14)

The 14 articles that classified HL as an SDoH did so following the World Health Organization’s classification and previous research or by defining determinants as factors that impact or predict health outcomes. Aaby et al [45] classify HL as an SDoH because it is a combination of “personal competencies and situational resources” that affects individuals’ interaction with health care institutions. Some authors, despite describing HL as an SDoH, still note that it is related to other SDoH as well. Cheuhuen et al [47] identify that HL is associated with economic stability and education, and Lee et al [55] and Sentell et al [46] both associate it with the social context. Articles also identified various health outcomes that HL may impact. Cabellos-Garcia et al [48] and Zhou et al [57] identified that poor HL could lead to reduced understanding of disease conditions and engagement with providers. Nevertheless, all 14 articles emphasize that HL is a modifiable SDoH that can change over time through interventions [14,45-57].

#### HL as a Mediator of SDoH (n=6)

A total of 6 articles established that, as a mediatory variable, HL can both compensate for and contribute to disparities in SDoH. Some articles define HL as an SDoH itself but further classify it as a mediator for other determinants. All 6 articles included education and income as SDoH for which HL could serve as compensation [58-63]. Bennett et al [59] also suggest that having high HL can compensate for racial or ethnic disparities in health outcomes. Zanchetta et al [63] describe that HL mediates between disparities in health care access and quality as well as social cohesion and context. To address poor HL among patients, van der Heide et al [62] recommend simplifying medical jargon. When designing these interventions, Bennett et al [59] emphasize considering complex patient perspectives and unique demographic needs, such as those of the geriatric population, which differ from those of younger patients.

#### Informatics Interventions

The secondary objective of the study was to identify informatics-based interventions to improve HL. Articles rarely provided specific intervention recommendations but instead listed several potential problems, such as complicated medical jargon or low health awareness, that complicate patient understanding of health information. Therefore, 4 informatics-based solutions were proposed based on the research team’s knowledge and experience for the identified problems, as follows: (1) language or text simplification, (2) population-focused (or policy-based) interventions, (3) health education efforts, and (4) patient identification. Since the included articles were largely not interventional in nature, the following sections extrapolate on the recommendations with references to ongoing studies that have implemented these strategies.

## Discussion

### Principal Findings

This systematic review included 43 papers and reported the results following the PRISMA guidelines. Most studies were conducted in Europe in the past 5 to 10 years. The studies examined HL in relation to the two themes of SDoH—health-focused and demographic—and generated 3 roles for HL, as follows: a mediator of SDoH, a result of SDoH, and modifiable SDoH. More than half of the studies had a cross-sectional design. However, HL is a complex, actionable variable that may be targeted by various strategies.

### Proposed Interventions

As clinical note sharing becomes more popular, generating interventions that address low HL becomes even more crucial. In this vein, we generated 4 recommendations for focused HL interventions based on the key findings of this systematic review.

Interventions with an informatics focus could play a particularly vital role in improving patient comprehension of health information as the health care field becomes increasingly mobile and technology dependent. It is important to consider experimental methods to measure the efficacy of implementing these strategies. Including control groups and validated HL measuring tools can help monitor how different interventions influence patient HL levels. Validated measuring tools include the Rapid Estimate of Adult Literacy in Medicine (REALM), REALM-Short Form, Short Assessment of Health Literacy-Spanish and English (SAHL-S&E), Brief Health Literacy Screen (BHLS), and Test of Functional Health Literacy in Adults (TOFHLA) [64-66]. The REALM, REALM-SF, and SAHL-S&E have all been validated and recommended by the AHRQ. The REALM and SAHL-S&E are recommended for research purposes to assess participant HL, while the REALM-SF, BHLS, and TOFHLA have been validated for use in screenings in clinical settings [66,67]. The REALM-SF is particularly designed to identify limited literacy levels [67]. Therefore, the clinically usable metrics may be more relevant for interventions that take place in health care settings, such as patient identification.

#### Language and Text Simplification

Text simplification addresses the tendency of clinic notes and health information in general to include medical jargon that exceeds the comprehension levels of most patients [42,44,62]. Even patients with highly educated backgrounds have shown low scores on HL surveys. Therefore, text simplification can benefit patients across all HL competencies by reducing jargon and making health information more easily understandable and usable [62]. Text simplification does not replace the existing clinic note shared between providers; it provides a simplified version for patients in addition to the original note. Current research indicates that the most effective manner of text simplification relies on manual editing techniques using human oversight of a text simplification process, combined with information visualization [68]. Although simplification improves patient comprehension, manual editing could strain health care professionals’ workload. Therefore, developing informatics interventions that automate text simplification while retaining the grammatical and logical integrity of the clinical text is important. Current automated simplification methods scored poorly due to grammatical errors, repetition, and inconsistencies in the autogenerated documents [68]. Artificial intelligence–derived text simplification methods may overcome these barriers by matching a document’s reading level to the readers’ needs, as shown in a study where ChatGPT was able to modify answers to men’s health condition questions to accommodate lower reading levels [69,70]. However, popularly used AI tools, such as ChatGPT, need considerable evaluation to minimize inaccurate information delivery and improve comprehensibility. Current studies indicate that these tools lack citations for the information they provide and cannot differentiate between low-quality and high-quality information [70,71].

#### Population-Based Visualization and Cross-Cultural Communications

HL needs are different across populations and cultural contexts, and interventions should account for these differences. For example, non–English-speaking individuals are overlooked in many HL studies, and interventions targeting English speakers will not always suit those with a limited or nonnative grasp of English [23,72]. Realizing this limitation, the OPHELIA (OPtimising HEalth LIterAcy) [73] project is a multisite study that assesses HL strengths and weaknesses in their patient population at each study site and uses these responses to determine appropriate intervention methods. Equally important is including representatives from the community in intervention design. A systematic review looking at interventions that address HL among Aboriginal and Torres Strait Islander community members noted that many failed to include these patients in the design process and consequently had limited participant retention [74]. Another facet is implementing policy-level changes that increase access to HL support. This is particularly relevant for patients who face health inequity. However, implementing these changes has been slow. In the European Union, challenges such as funding constraints and obstacles to initiatives have prevented effective execution beyond a few countries [75]. The population-based and policy-level interventions should consider visual analytics to explore meaningful patterns in a large data set and use recent advances in natural language understanding and translation to promote cross-cultural communication [76].

#### Patient Identification

Although population-focused and policy-level interventions address low HL at the macro level, such methods may overlook the individual HL needs of a patient. Therefore, screening HL levels as a part of standard practices in health care settings can help identify patients who need additional support at the clinic visit and can expedite provider response [36]. For example, Vanderbilt University Medical Center and the University of Arkansas Medical Sciences incorporate HL screening as part of their educational health assessment and have done so since 2010 and 2016, respectively [77]. Screening may also involve various informatics tools. For example, patients can be actively screened using electronic data capture tools (eg, REDCap) [78]. These informatics tools should be integrated into clinical workflow to ensure the quality of data. On the other hand, patient cohorts can be identified by reports or dashboards of electronic health records or medical text search engines (eg, Electronic Medical Record Search Engine [EMERSE]) [79]. Once the patient group is targeted, inclusive HL interventions can be designed and executed. However, implementing screening practices should be done with caution to avoid perpetuating stigma or embarrassment. Integrating screening questions within the clinical workflow and training health professionals on screening administration can help address these concerns [77].

#### Health Education and Online Community Building

Given the relevance of socialization and environment on HL development, it is important to consider interventions that cultivate HL through health education. Health care providers, such as nurses and community health workers, have important roles in providing education and reinforcing patient understanding of their health conditions [63,80]. However, the burden on health education cannot be placed on providers alone. Health education programs implemented by health care organizations and community health centers can actively and effectively improve HL [81]. It is important to adapt these programs for cultural and demographic sensitivity and patient-provider communications. For example, a recent study targeting older adult needs emphasized the need to include the patient’s caregivers and to accommodate barriers in comprehension, especially cognitive ones [82]. Health education intervention should consider developing an online community, such as ImproveCareNow, to promote collaborative care and build repositories of patient education materials with well-designed education programs to help patients improve their HL [83]. Including the input of individuals who are well-integrated into and familiar with the needs of a patient population, such as community health workers, can also be helpful in this process [80].

### Limitations

There are a few limitations in the methodology and generalizability of our research. First, we conducted a database search of only PubMed and Scopus, limiting the scope of the article search. However, PubMed and Scopus are two of the most popular and largest databases in biomedical and social science research. During the analysis, it was clear that the results were concise and supported one another. For example, several articles noted multiple roles for HL but tended to focus on one. Second, very few articles included noncorrelated results because of their cross-sectional designs. This prevented researchers from drawing a causative relationship between HL and SDoH, but they nevertheless had hypotheses for relationships that informed our classification. Third, the PRISMA guidelines were updated in 2020 with new standards and recommendations for systematic reviews. As we had already made considerable progress in this project before the revision was published in 2021, we completed the data analysis using the 2015 reporting standards that originally informed our methods. However, in cross-referencing our methods with the 2020 revisions, our research largely adheres to the new guidelines [84].

### Conclusions

The articles included in this literature review indicate that HL can adopt various roles in conjunction with SDoH. This flexibility makes HL an appropriate topic for intervention to accommodate poor health outcomes and improve patient autonomy. However, the complex nature of HL means that it warrants further research to understand how HL-targeted interventions impact this process.

## Appendix

Multimedia Appendix 1 Literature search and information extraction.

Multimedia Appendix 2 PRISMA Checklist.

## Abbreviations

AHRQ: Agency for Healthcare Research and Quality
BHLS: Brief Health Literacy Screen
EMERSE: Electronic Medical Record Search Engine
HL: health literacy
OPHELIA: OPtimising HEalth LIterAcy
PRISMA: Preferred Reporting Items for Systematic Reviews and Meta-Analyses
RCT: randomized clinical trial
REALM: Rapid Estimate of Adult Literacy in Medicine
SAHL-S&E: Short Assessment of Health Literacy-Spanish and English
SDoH: social determinants of health
TOFHLA: Test of Functional Health Literacy in Adults
